# Microcephaly in Neurometabolic Diseases

**DOI:** 10.3390/children9010097

**Published:** 2022-01-11

**Authors:** Wiktoria Kempińska, Karolina Korta, Magdalena Marchaj, Justyna Paprocka

**Affiliations:** Students’ Scientific Society, Department of Pediatric Neurology, Faculty of Medical Sciences in Katowice, Medical University of Silesia, 40-752 Katowice, Poland; wikikem@interia.pl (W.K.); karolina.korta@onet.pl (K.K.); magdalenamarchaj@interia.pl (M.M.)

**Keywords:** microcephaly, neurometabolic, neurometabolic diseases, congenital microcephaly, acquired microcephaly

## Abstract

Neurometabolic disorders are an important group of diseases that mostly occur in neonates and infants. They are mainly due to the lack or dysfunction of an enzyme or cofactors necessary for a specific biochemical reaction, which leads to a deficiency of essential metabolites in the brain. This, in turn, can cause certain neurometabolic diseases. Disruption of metabolic pathways, and the inhibition at earlier stages, may lead to the storage of reaction intermediates, which are often toxic to the developing brain. Symptoms are caused by the progressive deterioration of mental, motor, and perceptual functions. The authors review the diseases with microcephaly, which may be one of the most visible signs of neurometabolic disorders.

## 1. Introduction

Microcephaly is a developmental malformation characterized by decreased cranial size. Microcephaly is diagnosed by measuring the occipitofrontal circumference (OFC). It is measured between the region above the supraorbital ridges and the most prominent part on the back of the head. Microcephaly is diagnosed when a head circumference is below the third percentile or is more than two standard deviations (SDs) below the mean adjusted for age and sex. Some researchers were in favor of defining severe microcephaly as a head circumference > 3 SDs below the mean [1,2,3]. Individuals with microcephaly have a brain weight of less than 900 g. Microcephaly can occur as an isolated defect or as part of the syndrome of defects. Relative microcephaly is characterized by a proportional reduction in all parameters of physical development (body weight/length, and head circumference). In absolute microcephaly, the head circumference is significantly different from body weight and length, which are within normal ranges. In clinical practice, absolute microcephaly significantly more often occurs with the features of intellectual disability and neurological disorders. However, these problems can occur in all cases of microcephaly because the head circumference is usually a reflection of brain size. Microcephaly is estimated at between 2 and 12 children per 10,000 births [2]. 

The causes of microcephaly are divided into genetic and non-genetic. The most common non-genetic causes include hypoxic-ischemic encephalopathy/bleeding to the central nervous system (CNS), trauma to the CNS, effects of chemicals (e.g., alcohol) or drugs (e.g., antiepileptic drugs), maternal diseases during pregnancy (e.g., diabetes, phenylketonuria), and intrauterine infections (e.g., varicella, cytomegalovirus, rubella, and toxoplasmosis). Additionally, it was proved that Zika Virus Syndrome (ZIKV) infection in pregnancy is associated with congenital microcephaly [2,4].

Division of microcephaly into congenital (primary) and acquired (secondary) is useful for determining the etiology, symptoms, and prognosis (Table 1). Primary microcephaly occurs no later than at 32 weeks’ gestation and is probably caused by a reduced number of neurons formed during neurogenesis. This form of microcephaly is an effect of inhibition of normal brain development at its early stage. Secondary microcephaly, whose onset is defined at different time intervals, occurs usually at about 32 weeks’ gestation, is presumably due to decreased dendritic activity (or formation of dendritic processes) of neurons while their normal number is preserved. Secondary microcephaly generally results in a neurodegenerative process in the CNS and may occur in inborn errors of metabolism. Environmental factors probably play a significant role in the etiopathogenesis of secondary microcephaly. There are also distinguished isolated microcephaly and syndromic microcephaly (which coexists with dysmorphic features and/or congenital defects what affect other organs) [2]. 

Assessment of children with microcephaly is related to obtaining a thorough history, clinical examination, and further follow-up tests. MRI is usually the method of choice in the search for the etiology of microcephaly. Prenatal infections are the most common etiology. However, the cause remains unknown in 50% of cases.

This paper presents microcephaly as a clinical manifestation in the course of neurometabolic diseases. The purpose of our review is to present the diseases in which microcephaly may occur, which are increasingly common problem.

## 2. Congenital Microcephaly

### 2.1. CK Syndrome—X-Linked Syndromic Intellectual Disability Disorder Characterized by Thin Body Habitus and Cortical Malformations, OMIM 300831

CK syndrome is an X-linked syndromic intellectual disability disorder characterized by thin body habitus and cortical malformations. The syndrome is caused by a mutation in the NSDHL gene encoding NAD(P) dependent steroid dehydrogenase-like, which is involved in cholesterol biosynthesis. The NSDHL gene is located on the X chromosome (locus Xq28). It is an X-linked recessive inheritance trait. The disease affects mostly males [5]. Precise prevalence of the disease is unknown.

The characteristic features include asthenic body build, congenital microcephaly, delayed psychomotor development, intellectual disability, early-onset epileptic seizures, speech delay, cortical malformations, attention deficit hyperactivity disorder, aggression, and irritability. Dysmorphic features such as an elongated face, posteriorly rotated ears, epicanthal folds, almond shaped eyes, micrognathia, high nasal bridge, high arched palate, relatively long, thin fingers, and toes may also be present. Other symptoms include hypotonia, strabismus, kyphosis, scoliosis, and dental defects [5]. Serum and CSF cholesterol level may be normal. Mc Larren et al. (2010) suggested that methyl sterol accumulation can participate in pathogenesis of the disease [5]. 

### 2.2. Asparagine Synthetase Deficiency (ASD), OMIM 615574

Asparagine synthetase catalyzes the synthesis of asparagine from aspartate and glutamine in an ATP-dependent reaction [6]. Asparagine synthetase deficiency is an autosomal recessively inherited disorder that is caused by a mutation in the ASNS gene (locus 7q21.3) [6,7]. The loss of function of the asparagine synthetase leads to cortical atrophy with reduced brain volume and pachygyria. By 2020, fewer than 40 cases of mutation in *ASNS* gene have been reported [8]. 

ASD typically manifests as congenital microcephaly, psychomotor retardation, axial hypotonia and appendicular hypertonia. Many affected individuals develop epileptic seizures (tonic–clonic, myoclonic, or tonic seizures). The disease is clinically manifested by growth retardation, feeding difficulties (e.g., gastroesophageal reflux) and cortical blindness. Dysmorphic features include micrognathia, brachycephaly, large ears, and pear-like head shape [6,7,8,9].

The level of asparagine in the cerebrospinal fluid (CSP) can be normal, but also low. Plasma asparagine level is reduced or in normal range [6,7,8,9]. 

Neuroimaging studies show cerebral atrophy, simplified gyral pattern, hypoplastic cerebellum, cortical atrophy, delayed myelination, ventriculomegaly, and hypoplasia of the pons [6,7,8].

### 2.3. Neu–Laxova Syndrome, OMIM NLS1-256520; NLS2-616038

Neu–Laxova syndrome (NLS) is a lethal syndrome of intrauterine growth retardation and multiple malformations. The condition is most likely caused by mutations in the PHGDH, PSAT1, or PSPH genes, which are associated with the serine synthesis pathway [10,11]. Neu–Laxova syndrome type 1 is a more severe phenotypic variant of 3-PGDH deficiency. The mutation in the PHGDH gene (locus 1p12) occurs. Most PHGDH mutations that cause NLS are missense mutations, although nonsense, splicing or frame shift mutations have also been reported [11]. Acuna-Hidalgo et al., (2014) reported close parental consanguinity in few investigated families [10]. Prenatal ultrasonography can help to diagnose the disease [12,13]. Approximately 75 cases of NLS have been reported in the literature [14].

#### 2.3.1. NLS 1

The characteristic features of NLS 1 include prenatal growth retardation, congenital microcephaly, CNS abnormalities such as lissencephaly, hypoplasia or absence of the corpus callosum, cerebellar hypoplasia, aplasia of olfactory structures and the optic nerves, spina bifida, Dandy–Walker malformation, ventriculomegaly, and choroid plexus cysts. Micrognathia, large, low-set ears, hypertelorism, exophthalmos, and lack of eyelids and eye lashes are often found in patients. Ectropion, cataract, flattened nose, slanted forehead, cleft lip, and cleft palate are also characteristic. Abnormalities of internal organs such as an atrial and ventricular septal defect, transposition of the great arteries, persistent ductus arteriosus, pulmonary hypoplasia, kidneys disorders (e.g., unilateral absence of kidney), and genital abnormalities are also common. There is also increased swelling of the subcutaneous tissue, ichthyosis, muscular atrophy, permanent joint contracture, syndactyly, rocker bottom feet, shortened neck, and short limbs [10,11,12,13,14]. Features of NLS such as microcephaly, short and broad neck, and generalized skin edema may be detected during fetal ultrasound [13]. 

#### 2.3.2. NLS 2

NLS type 2 is an autosomal recessive disorder caused by a mutation in the PSAT1 gene (locus 9q21.2) encoding phosphoserine aminotransferase 1, enzyme catalyzing the conversion of 3 phosphohydroxypyruvate into 3-phosphoserine [10]. Acuna-Hidalgo et al. (2014) reported patients with Neu–Laxova syndrome-2 [10]. 

Treatment with serine and glycine supplementation can be successful in PSAT1 deficiency if therapy is started immediately after birth [10].

### 2.4. Maternal Phenylketonuria, OMIM 261600

Maternal phenylketonuria is a group of congenital malformations that occur in children of mothers with untreated hyperphenylalaninemia or phenylketonuria (an autosomal recessively inherited disorder of phenylalanine metabolism caused by the mutation in the phenylalanine hydroxylase (PAH) gene) [15,16,17]. PAH deficiency is common in Caucasians in whom the overall incidence is 1 in 10,000 live births, it is particularly common in Ireland and Turkey where the incidence is 1 in 4500 and 1 in 2600, respectively [15].

Excess phenylalanine hinders the transport of other amino acids across the placenta and is accumulated in the fetal CNS, which causes neurological disorders and intellectual disability. “Microcephaly is the most common fetal malformation associated with elevated maternal PHE levels during gestation” [15]. Other common symptoms are intellectual disability, low birth weight, congenital heart defects, behavioral disorders, motor hyperactivity, difficulties related to learning, planning, speech, and memory [15,17].

### 2.5. Microcephaly, Amish Type (MCPHA), OMIM 607196

This is an autosomal recessive disorder with primary progressive microcephaly. At birth, the head circumference is usually -4SD to -12SD [18]. The disease has been found among the Amish people of Pennsylvania, with an estimated frequency of 1 in 500 births [19]. It is caused by mutations in the SLC25A19 gene located on chromosome 17, which encodes a mitochondrial deoxynucleotide carrier in the ATP-dependent process. This results in a decrease in the rate of mitochondrial DNA synthesis, which, in turn, leads to energy deficits and impaired brain growth [18].

In addition to severe primary microcephaly, patients have distorted facial features, a drooping forehead, and a small anterior fontanel. They are also characterized by severely retarded psychomotor development and intellectual disability. Such patients present with myoclonus and axial hypotonia with increased limb muscle tone [18,19].

Laboratory findings show elevated blood levels of alpha-ketoglutaric acid, and lactic acidosis may also be present [18,19].

The symptomatic and maintenance therapy is used in the affected patients. Acidosis is controlled by a high-fat diet [18].

### 2.6. Hyperphenylalaninemia with BH4 Deficiency Due to GTPCH Deficiency, OMIM 233910

GTPCH deficiency is caused by a mutation in the GCH1 gene (locus 14q22.2) encoding GTP-cyclohydrolase I [20,21]. GTPCH is involved in the biosynthesis of folic acid and biopterin, which causes tetrahydrobiopterin deficiency in the case of the dysfunction of this enzyme. Tetrahydrobiopterin deficiency affects the occurrence of hyperphenylalaninemia and is also responsible for defective monoamine neurotransmission due to the impaired function of tyrosine and tryptophan hydroxylases, which are tetrahydrobiopterin-dependent hydroxylases [20,21]. It is an autosomal recessive inherited disorder. The precise global prevalence of GTPCH deficiency remains unknown [21]. 

The common symptoms of this syndrome include feeding difficulties in infancy, hypersalivation, and dysphagia. In severe cases, GTPCH deficiency may result in the occurrence of microcephaly [20,21,22]. 

The following symptoms are also reported: psychomotor retardation, axial hypotonia, limb muscle hypertonia, seizures, dystonia, oculogyric crises, choreoathetosis, tremor, a reduced response to stimuli, and episodic, recurrent hyperthermia (caused by dysregulation of the autonomic nervous system) [20,21]. 

Laboratory findings show hyperphenylalaninemia, decreased homovanillic acid (HVA), 5-hydroxyindoleacetic acid (5-HIAA), neopterin and biopterin in CSF, decreased neopterin and biopterin in urine [21]. 

Treatment includes a low-phenylalanine diet, oral BH4, the administration of carbidopa, L-DOPA, and 5-hydroxytryptophan [20,21].

### 2.7. Dihydropteridine Reductase Deficiency (Phenylketonuria Type 2), OMIM 261630

Dihydropteridine reductase (DHPR) deficiency is a rare autosomal recessive inheritance disorder in which tetrahydrobiopterin (BH4) metabolism is impaired. It causes hyperphenylalaninemia and neurotransmitter deficiency. The mutation is related to the QDPR gene (locus 4p15.32) [21,23]. The incidence of all causes of hyperphenylalaninemia detected by newborn screening programs is estimated to be approximately 1:10,000 (data for Europe). BH4 deficiencies constitute around 1–2% of these cases. DHPR deficiency accounts about 33% of BH4 deficiencies [21].

Neurological syndromes due to hypomyelination in the CNS are diagnosed. Individuals with DHPR deficiency may present with microcephaly, intracerebral calcifications, axial hypotonia, and hyperreflexion, developmental delay, and seizures. In some cases, hypertonia and dyskinesis can occur. Feeding problems in infancy, dysphagia, hypersalivation, and irritability are also reported [21,22,23,24].

Laboratory abnormalities include hyperphenylalaninemia, decreased homovanillic acid (HVA) and 5-hydroxyindoleacetic acid (5-HIAA) in CSF, and increased biopterin in urine and CSF. The disease is usually suspected due to an elevated concentration of phenylalanine during newborn screening. The diagnosis is made by measuring decreased DHPR activity in blood cells [21,23,24].

The aim of therapy is to lower blood phenylalanine levels using BH4 supplementation, which is mostly combined with a phenylalanine-restricted diet. Treatment with neurotransmitter precursors (5-hydroxytryptophan and L-DOPA/carbidopa) is an important part of therapy. Long-acting dopamine agonists (e.g., pramipexole), monoamine oxidase inhibitors (e.g., selegiline), or benserazide can also be used. In the absence of treatment, a progressive neurological deficit occurs [21,23,25].

### 2.8. Smith–Lemli–Opitz Syndrome (SLOS), OMIM 270400 

Smith–Lemli–Opitz syndrome (SLOS) is a genetic metabolic disease of autosomal recessive inheritance caused by a mutation in the DHCR7 gene (locus 11q13.4) encoding 7-dehydrocholesterol reductase. The prevalence of SLOS is estimated to be approximately 1:20,000 to 1:60,000 [26]. 

SLOS is characterized by high variability of the clinical picture. The most severe form of SLOS is lethal, whereas mild forms often remain undiagnosed. The clinical picture is characterized by many dysmorphic features (e.g., low set and posteriorly rotated ears, short nose with anteverted nares, and hypertelorism), microcephaly, cleft palate, underdeveloped external genitalia in males, hypospadias, cryptorchidism, polydactyly, and syndactyly of digits two and three. Psychomotor retardation and (moderate to profound) intellectual disability are characteristic features of the syndrome. During infancy, gastrointestinal disorders can occur (constipation, vomiting, problems with sucking, and pyloric stenosis), which results in decreased weight gain. Muscle hypotonia, seizures, eye abnormalities (e.g., cataract), ptosis, hearing loss, and inguinal hernia are also common. Renal defects are also of common occurrence and include hydronephrosis, renal agenesis, and cysts. Cardiovascular symptoms are also reported (ventricular and atrial septal defect, patent ductus arteriosus). In the later period, learning difficulties and behavioral disorders such as aggression or self-mutilation are also reported. Disorders from autism spectrum can occur [26,27,28].

The diagnosis of the disease is established in individuals with typical clinical features and elevated serum concentration of 7-dehydrocholesterol. Additionally, it can be established by the identification of pathogenic variants in DHCR7 using molecular genetic testing. Serum cholesterol level is usually low. However, it may be within the normal range in about 10% of patients [26].

Dietary cholesterol supplementation is the common form of treatment for SLOS. By affecting HMG-CoA reductase, statins regulate the rate of cholesterol synthesis pathway. Inhibition of the pathway helps to reduce the accumulation of toxic metabolites such as 7DHC. The use of antioxidants is also considered. Tauroursodeoxycholic acid, which is a bile acid with antioxidant, antiapoptotic, and neuroprotective properties has potential therapeutic properties [26,29,30].

## 3. Acquired Microcephaly

### 3.1. Neurodevelopmental Disorder with Progressive Microcephaly, Spasticity, and Brain Imaging Abnormalities (NEDMISBA), OMIM 616486

Docosahexaenoic acid (DHA) has an essential structural role in neuronal cell membranes in the brain. DHA is a component of membrane phospholipids at the sn-2 position of phosphatidylethanolamine (PE) and phosphatidylserine (PS). DHA is also a precursor of docosanoids and elovanoids [31]. Mfsd2a is the main DHA transporter across the blood–brain and the blood–retinal barriers. It transports DHA bound to lysophosphatidylcholine [31]. The transporter is encoded by the MFSD2A gene (locus 1p34.2). Clinically, it is manifested by the presence of two mutant alleles (autosomal recessive inheritance) [32].

Neurodevelopmental disorder with progressive microcephaly, spasticity, and brain imaging abnormalities caused by the mutation in the transporter for DHA has a diverse phenotype. The mutation in the transporter results in DHA deficiency in the brain, which may manifest in the fetal life in the form of hydrocephalus, enlargement of the ventricular system (especially lateral ventricles), cerebellar and brainstem hypoplasia and aplasia, and thinning of the corpus callosum. After birth, the clinical picture of the disease includes progressive microcephaly, spastic paralysis, epileptic seizures, psychomotor retardation, and intellectual disability [22,32,33,34]. Cases of muscular hypotonia, dystonia have also been reported. The disease is characterized also by feeding difficulties, speech disorders, strabismus, and facial dysmorphia (e.g., tented upper lip, and wide nasal bridge with epicanthal folds) [32,33,34]. 

Laboratory studies show increased plasma lysophosphatidylcholine (LPC) levels containing mono- and polyunsaturated fatty acyl chains [33].

### 3.2. De Vivo Disease, OMIM 606777

GLUT1 deficiency syndrome 1 is a genetic disorder which is caused by the mutation in the SLC2A1 gene (locus1p34.2) encoding the GLUT1 glucose transporter protein [35,36,37]. Autosomal dominant and autosomal recessive inheritance is reported [36]. GLUT1 deficiency adversely affects the development and function of the brain microvascular network, which has abundant expression of receptors in endothelial cells of the brain microvasculature, where GLUT1 protein participates in the transport of blood glucose across the blood–brain barrier into the CNS [35]. In retrospective studies, the prevalence of Glut1DS was estimated to be 1:83,000 in Denmark 5 and 1:90,000 in Australia; however, recent researches suggest a more frequent occurrence of the disease [36].

The diagnosis of GLUT1 deficiency is established in individuals with typical clinical symptoms, normal blood glucose levels, cerebrospinal fluid glucose levels < 40 mg/dL, and the identification of a heterozygous pathogenic variant in SLC2A1 [36,37]. The hallmarks of De Vivo disease include early-onset, epileptic seizures, delayed development, ataxia, and dystonia. Acquired microcephaly, spasticity, choreoathetosis, psychomotor developmental disorders, speech disorders (speech retardation, and dysarthria), and learning difficulties are also reported [35,36,37]. 

Laboratory findings show hypoglycorrhachia (CSF glucose < 40 mg/dL, glucose index < 0.45) and low to normal CSF lactate levels are typical. A favorable response to a ketogenic diet is reported [36,37].

### 3.3. Neurodevelopmental Disorder with or without Hyperkinetic Movements and Seizures Due to NMDA Receptor Dysfunction-NDHMSA, OMIM 614254

The *GRIN1* gene encodes the GluN1 protein, the primary subunit of the N-methyl-D-aspartate (NMDA) receptor, which is a heteromeric glutamate-gated ion channel essential for synaptic functions in the brain [38,39]. NDHMSD is an autosomal dominant inherited disorder (mutation at locus 9q34.3). However, autosomal recessive cases of *GRIN1-*NDD also have been reported (OMIM 617820). Fewer than 100 cases of GRIN1-NDD have been described [40].

Symptoms occur in infancy. Some patients may present with microcephaly, cortical blindness, oculogyric crisis, early-onset, epileptic seizures, delayed psychomotor development, severe intellectual disability, spasticity, and inability to walk. Speech disorders or complete absence of speech are also common. Symptoms suggestive of extrapyramidal system dysfunction include hyperkinetic movements, dyskinesia, myoclonus, choreic movements, severe muscle hypotonia and stereotypic movements. Aggression, sleep disorders, autistic features, self-mutilating behaviors are also characteristic. Gastrointestinal symptoms such as feeding problems are also present. The disorder should be suspected in individuals with typical clinical symptoms and typical MR imaging, including cerebral atrophy, ventriculomegaly, and thinning of the corpus callosum [38,39,40,41].

### 3.4. Krabbe Disease (Globoid Leukodystrophy), OMIM 245200

Krabbe disease is a rare disease with an estimated incidence of approximately 1:100,000 births [42]. Globoid leukodystrophy is caused by mutations in the GALC gene at locus 14q31.3 encoding β-galactocerebrosidase. The inheritance is autosomal recessive [42]. Galactosylceramidase is essential for the breakdown of galactolipids, mainly galactosylceramide and galactosylsphingosine. Accumulation of unmetabolized neurotoxic substrates leads to demyelination of the CNS and peripheral nervous system. Krabbe disease has different phenotypes [42,43].

The early infantile subtype is the most prevalent phenotypic variant of this disease (about 85% of patients with infantile type of the disease) [43]. It is characterized by onset before the age of 1 year, severe psychomotor delay or regression, and premature death [42,43]. Late infantile Krabbe disease accounts for approximately 20% of infantile cases. It occurs between 13 and 36 months. Its course is slower than the early infantile subtype. It is characterized by psychomotor regression, spasticity, irritability, and gait abnormalities [43]. The less frequent forms include the juvenile subtype (occurring between 3 and 16 years of age) and the adult subtype (after 16 years of age) [42].

Typical symptoms of globoid leukodystrophy include delay of psychomotor development, deafness (frequent abnormal auditory brainstem responses (ABR)), blindness (abnormal visual evoked potentials (VEP)) and feeding difficulties. The disease may manifest with microcephaly, appendicular spasticity, axial hypotonia, abnormal deep tendon reflexes, and epileptic seizures. Peripheral neuropathy is also characteristic. EMG examination shows slowing of nerve conduction [42,43,44,45].

Krabbe disease is suspected based on the clinical picture and MR imaging which shows progressive white matter abnormalities (the most common periventricular changes and in the centrum semiovale) [43]. Cerebellar pathology, involving the dentate nucleus can occur [45]. The diagnosis is established based on enzymatic studies, which mostly show GALC deficiency or a reduced activity of GALC in leukocytes and fibroblast cultures. An elevated protein level in CSF is also reported. Genetic analysis of the mutation confirms the diagnosis [42,43]. 

Treatment consists of transplantation of hematopoietic stem cells (HSC) collected from bone marrow or cord blood. The most benefits from transplantation are derived if the therapy was applied to the asymptomatic patients. Unfortunately, patients with symptomatic early infantile subtype have outcomes comparable to those of untreated patients [43].

### 3.5. Menkes Disease (Curly Hair Disease), OMIM 309400

Menkes disease (MNK) is a genetically determined neurodegenerative disorder related to the inability to metabolize copper. It is caused by the mutation in the ATP7A gene at locus Xq21.1, which encodes a Cu2+-transporting ATPase. The inheritance is X-linked recessive [46,47]. The incidence of the disease is about 1 per 300,000 live births [47].

CNS symptoms include neurodegeneration, muscle hypotonia, intellectual disability, seizures (due to NMDA receptor overactivity), ataxia, and intracranial hemorrhages. The characteristic features of the disease also include hypothermia, pudgy cheeks, osteoporosis, wormian bones, joint laxity, hypopigmentation and skin laxity, and kinky, steel-colored, thinned hair. The retardation of growth, poor weight gain, and microcephaly can occur [46,47,48]. Symptoms related to autonomic dysfunction are also present (chronic diarrhea, orthostatic hypotonia, and syncope). The classic severe form with therapy-resistant seizures usually occurs at 2–3 months of age. Death usually occurs before the age of 3 years [46,47]. A low plasma level of copper and ceruloplasmin are characteristic of MNK [47]. Deficiency of many copper-binding enzymes (e.g., superoxide dismutase, lysyl oxidase, cytochrome c oxidase, tyrosinase, and sulfhydryl oxidase) is observed [46]. Brain imaging shows diffuse cerebral and cerebellar atrophy, white matter changes. Moreover, subdural hygromas can be visualized on imaging [47]. 

The aim of targeted MNK treatment is to supply copper to tissues and copper-dependent enzymes. Oral copper administration is ineffective because copper is not absorbed in the gut. It should be supplemented parenterally or subcutaneously. Of the available copper compounds, copper histidine (copper–histidine complex) is mostly used. The benefits of copper and histidine supplementation depends on early treatment and the presence of at least partially functional ATP7A [46].

### 3.6. Cerebral Folate Deficiency (CFD), OMIM 613068

Folic acid (vitamin B9) plays several roles in the body, particularly in the brain. It assists in the metabolism of purines and pyrimidines, which are the building blocks of RNA and DNA and are essential for proper energy production, neurotransmitter production, cellular detoxification, and the proper formation of the nervous system during development [49]. 

Folic acid deficiency syndrome in the brain, also known as CFD, is a condition characterized by low levels of 5-methyltetrahydrofolate (5-MTHF) in CSF but a normal (or even elevated) level of 5-MTHF in the blood. Decreased 5-MTHF level in CSF results from the dysfunction of the folic acid receptor alpha (FR⍺). Impaired functioning of the FR⍺ most often results from the presence of autoantibodies blinding to the FR⍺, which disrupts its function, or results from a rare mutation in the FOLR1 gene, which can lead to an autosomal recessive genetic condition that disrupts FR⍺ function. The receptor becomes dysfunctional so that hardly any folic acid or MTHF can be properly transported to the brain, which leads to many neurological consequences [50,51,52]. Fewer than 20 individuals with CFD have been reported in scientific literature.

Initial symptoms of the disease, which may occur as early as 4–6 months of age, include irritability and trouble sleeping (insomnia). Over time, delayed psychomotor development can be noted, including hypotonia, hypokinesia, and abnormal head circumference growth. Between 4 months to 3 years of age, the following disorders may occur: epilepsy, gait and speech disorders, dyskinesia, cerebellar ataxia, and spasticity. In some cases, hearing loss and central vision abnormalities such as blindness and optic nerve atrophy may occur after 3 or 6 years of age, respectively [52]. Abnormalities typical of CFD include hypsarrhythmia in the EEG and decreased 5-MTHF level in CSF (normal folate levels may be present in serum and red blood cells). MR brain is usually normal. In some cases, loss of the white matter in the brain (leukodystrophy) may be observed. Frontotemporal atrophy and subcortical demyelination can occur after 18 months [53,54]. The test for the presence of two FR⍺ autoantibodies (blocking antibody; binding autoantibody) is performed to determine whether the autoantibodies are responsible for folate deficiency in the brain. Molecular genetic testing for mutations in the FOLR1 gene is available to confirm the diagnosis [54].

Treatment of CFD with oral leucovorin calcium (folinic acid) over an extended period of time can lead to a significant improvement in clinical symptoms and stabilization of 5-MTHF level in CSF. The earlier the treatment, the better the outcome. Therefore, the best treatment response is seen when folinic acid supplementation is started in early childhood. Folic acid supplementation is not recommended since it is associated with adverse effects such as epileptic seizures, as opposed to leucovorin (no serious side effects were reported) [53].

### 3.7. Rhizomelic Chondrodysplasia Punctata Type 1 (RCDP1), OMIM 215100 

Rhizomelic chondrodysplasia punctata type 1 (RCDP1) is a type of peroxisome biogenesis disorder. RCDP1 is a rare disease, with a prevalence of 1/100,000 [55]. Peroxisomal diseases are divided into two groups. The former is associated with defects in organelle biogenesis, whereas the latter is related to the deficiency of a single enzyme. 

RCDP1 is an autosomal recessively inherited disease included in the first group of peroxisomal diseases. RCDP1 is caused by a homozygous or compound heterozygous mutation in the PEX7 gene, which encodes the PTS2 receptor. Individuals with RCDP1 present with large tissue stores of branched-chain fatty acids such as phytanic acid. Additionally, decreased levels of plasmalogen, which affects bone growth, are reported [55,56]. 

The age of onset is important because the severity of the clinical phenotype depends on it. Patients with a severe phenotype have hardly any motor or cognitive skills, while patients with a milder phenotype suffer from severe intellectual disabilities but they are able to walk and have good verbal communication skills. RCDP1 is characterized by bone defects, including proximal shortening of the humerus and the femur. A lot of patients experience seizures. The affected individuals also have myoclonic jerks, a distinctive facial appearance and often develop joint contractures. Somatic growth impairment and near absence of developmental milestones are also common. In addition, many affected individuals develop congenital cataract [57].

The characteristic laboratory findings include:

- Decreased concentration of plasmalogens in erythrocytes;
- High levels of phytanic acid [57].

### 3.8. Congenital Glycosylation Disorder Type I, OMIM 212065

Congenital glycosylation disorder type I is an autosomal recessively inherited genetic disorder induced by a mutation in the phosphomannomutase 2 gene. The defect is related to the formation of the dolichol lipid-linked oligosaccharide and its transfer to the nascent protein, which results in defective glycosylation of glycoconjugates. The estimated prevalence of CDG in European and African American populations is 1/10,000. To date, fewer than 100 cases have been reported for most CDG types [58].

Microcephaly occurs in about half of patients. They have characteristic dysmorphic features (fusiform phalanges of the fingers, prominent labia majora, inverted nipples, symmetric fat accumulations, and lipodystrophy of the buttocks). They are also characterized by motor and intellectual retardation, hypotonia, ataxia, stroke-like episodes, peripheral neuropathy, and gastrointestinal complaints (diarrhea and vomiting) [59].

Isoelectric focusing of the transferrin isoforms is performed to diagnose glycosylation disorders. Laboratory findings show the presence of abnormal serum glycoproteins (i.e., glycoproteins lacking sialic acid, galactose, and N-acetylglucosamine), elevated aminotransferase activity, proteinuria, hypocholesterolemia, and hypoalbuminemia. Phosphomannomutase deficiency is reported in the liver, leukocytes, or fibroblasts. Additionally, patients present with decreased levels of copper, iron, and zinc [60].

### 3.9. Molybdenum Cofactor Deficiency (MoCoD), OMIM 252150

Molybdenum cofactor deficiency (MoCoD) is an extremely rare autosomal recessive neurometabolic disease caused by a homozygous or compound heterozygous mutation in the MOCS1 gene on chromosome 6p21. Four genes are involved in the production pathway of molybdenum cofactor, i.e., MOCS1, MOCS2, MOCS3, and GEPH. Therefore, *MOCS1* mutations lead to MoCoD of complementation group A (MOCODA) [61,62,63,64].

So far, the literature describes an atypical late-onset phenotype in 13 patients) [62].

The disease usually presents in infancy (on average 12.5 months of age). The median survival is 36 months.

Microcephaly is often present. Drug-resistant epileptic seizures, increased muscle tone and feeding difficulties mostly occur in the first few days of life. Next to the above, the most common clinical manifestations include facial dysmorphia (swollen cheeks, prominent forehead, deep-set eyeballs, large ears, and elongated palpebral fissures. An epileptic seizure is the earliest, most characteristic symptom of this disease. Death occurs in early childhood in the most severe cases [65].

Biochemical abnormalities are related to decreased serum uric acid and an increased urinary sulfate level, which are associated with complex deficiencies of xanthine dehydrogenase and sulfite oxidase, of which molybdenum is a cofactor. Additionally, the disorder is characterized by low homocysteine levels and an abnormal urinary purine profile (uric acid is replaced by xanthine). Diagnosis is confirmed by genetic testing, which is important due to the possibility of therapy in 33% of MoCoD patients with a mutation in the MOCS1 gene [66].

Symptomatic treatment is applied. In patients with MOCODA, intravenous administration of purified cyclic pyranopterin monophosphate (cPMP) can be used. Asa result, the already existing lesions in the brain do not regress. However, drug-resistant epileptic seizures resolve, and neurotoxic effects and further brain damage are reduced. MOCS1 gene therapy based on an expression cassette is currently under investigation [66].

### 3.10. Isolated Sulfite Oxidase Deficiency (ISOD), OMIM 272300

Sulfite oxidase is the final enzyme in the oxidative degradation pathway of sulfur-containing amino acids and catalyzes the conversion of sulfites to sulfates. Isolated sulfite oxidase deficiency (ISOD), is a genetically determined disease caused by a mutation in the SUOX gene (locus 12q13.2). Frequency is unknown, but the disease is very rare. Disorder of the degradation pathway of sulfur amino acids leads to profound neurological disorders (encephalopathy) because of the toxicity of metabolites to the brain. It is an autosomal recessive inheritance disorder [67]. 

Early-onset ISOD is characterized by the onset of epileptic seizures in the first few hours or days of life. The disease is manifested by abnormal, increased limb muscle tone and abnormal movements such as choreoathetosis and dystonia [67]. In later stages, progressive microcephaly and profound intellectual disability are noted [67,68]. Subluxation or dislocation of the lens is another characteristic symptom that can be found after the neonatal period. Apart from neurological symptoms, the following can also occur feeding difficulties. Children usually die within the first few months of life. 

Laboratory findings suggestive of ISOD include elevated urinary sulfite levels, elevated urinary thiosulfate and S-sulfocysteine and significantly reduced plasma levels of total homocysteine [67]. The absence of xanthinuria distinguishes ISOD from MoCoD [68]. Head MRI often shows: loss of gray-white matter differentiation and cystic encephalomalacia. Thinning of the corpus callosum, ventriculomegaly, and diffuse cerebral atrophy can be observed. 

Currently, no causal treatment is available for the underlying metabolic defect. Treatment is only symptomatic [67].

### 3.11. Pelizaeus–Merzbacher Disease (PMD), OMIM 312080

Pelizaeus–Merzbacher disease (PMD) is a CNS demyelinating disease which belongs to the group of hypomyelinating leukodystrophies. It is inherited as an X-linked recessive trait. The disorder is caused by a mutation in the PLP1 gene located on the long arm of the X chromosome (locus Xq22.2) [69,70]. Mutations can be of various types (point mutations, duplications, nonsense, and deletions) [69]. PLP1 gene encodes the proteolipid protein, one of the major proteins of the myelin sheath [69,71]. The incidence of PMD ranges from 1 in 90,000 to 1 in 750,000 live births [69].

The clinical features of PMD include microcephaly, hearing impairment (brain auditory-evoked responses can be abnormal in PMD), nystagmus, and visual dysfunction. CNS symptoms also include axial hypotonia, ataxia, dystonia, seizures, choreoathetosis, and speech disorders (e.g., scanning speech). After hypotonia, spasticity may occur. Regression of psychomotor development and intellectual disability variant degrees occur [48,69,70]. In PMD, dysmyelination or hypomyelination of the brain is reported. There are different types of the PMD: classic, conatal, and transitional. The prognosis is highly variable. Patients with connatal, the most severe form (type II) have severe hypotonia, extrapyramidal signs, and die very early. Patients with classic PMD (type I), which onset is at infancy, live longer (adolescence to young adulthood). Transitional PMD combines clinical features of both the classic and connatal forms [69]. MRI shows hyperintense white matter lesions on T2-weighted imaging [69,70].

No causal treatment is currently available. Therapy is mainly symptomatic and palliative. However, research is ongoing to investigate treatment that targets the molecular mechanisms responsible for PMD. Currently, PMD is interesting therapeutic and research target for neural stem cell and glial progenitor cell transplantation. Lonaprisan, an antagonist of the progesterone receptor, caused mRNA overexpression decrease, increase number of myelinated axons, and functional motor improvement in PLP overexpressing mice (Prukop et al., 2014) [69,72]. 

## 4. Congenital and Acquired Microcephaly

### 4.1. Methylenetetrahydrofolate Reductase Deficiency (Homocystinuria Due to MTHFR Deficiency), OMIM 236250

Methylenetetrahydrofolate reductase (MTHFR) is an enzyme that catalyzes the reduction reaction of 5,10-methylenetetrahydrofolate to 5-methyltetrahydrofolate (5-MTHF). The MTHFR gene encoding the enzyme protein is located on chromosome 1 (locus 1p36.22) [73,74]. Homocystinuria caused by MTHFR deficiency is inherited in an autosomal recessive pattern. More than 200 cases of methylenetetrahydrofolate reductase deficiency have been reported [75]. 

Mutation in the MTHFR gene in severe forms of the disease results in cerebral atrophy, ventriculomegaly, microcephaly, cerebellar atrophy, hydrocephalus, hypomyelination, and pontine hypoplasia. Progressive encephalopathy, axial hypotonia, delay of psychomotor development, apnea, seizures, feeding problems can occur. Juvenile or late onset forms of the disease can also present thrombotic events, peripheral neuropathy, or psychiatric disturbances [73,74,75,76].

Low methylenetetrahydrofolate reductase activity, homocysteinemia, homocystinuria, and low to normal plasma methionine concentration are characteristic of this disease [73,74,75]. Full MTHFR gene sequencing can confirm the suspected clinical diagnosis.

Treatment of MTHFR deficiency includes the intake of supplements of betaine, folic acid, vitamins B6 and B12, and methionine [73,74,75].

### 4.2. Methylmalonic Acidemia with Homocystinuria, OMIM 277400

Methylmalonic acidemia with homocystinuria is a congenital defect of vitamin B12 (cobalamin) metabolism. There are five groups of cobalamin metabolism defects (Cbl-C, Cbl-D, Cbl-F, Cb1-J, and Cbl-X), which lead to methylmalonic acidemia with homocystinuria. Cobalamin C (Cbl-C) defect is the most common inborn error of cobalamin metabolism [77,78]. It causes impaired conversion of dietary vitamin B12 to its two metabolically active forms (methylcobalamin and adenosylcobalamin) [77,78]. The mutation in the MMACHC gene (locus 1p34.1) is responsible for this subtype of the disease [78,79]. The condition is inherited in an autosomal recessive pattern [77]. An incidence of cblC ranging from 1:100,000 to 1:200,000 births has been estimated [80].

Methylmalonic acidemia has a wide clinical spectrum, ranging from a mild condition to severe fetal congenital defects. The disease onset can occur from early infancy to adulthood [77,78]. Patients with methylmalonic acidemia with homocystinuria may show symptoms of psychomotor retardation and seizures. Cobalamin C (Cbl-C) defect results in microcephaly, hypomyelination and ventriculomegaly [48,76,79,81]. Progressive neuronal degeneration and hydrocephalus are reported. Extrapyramidal symptoms, delayed motor development, and hypotonia also occur. Patients with the Cbl-C type present with retinal degenerative changes (e.g., pigmentary retinopathy), which results in decreased visual acuity. Nystagmus and maculopathy may also occur. The appearance of patients with methylmalonic acidemia with homocystinuria is characterized by an elongated face and large low-set ears. The clinical picture may also show: poor feeding and dehydration in infancy, vascular changes (thrombotic microangiopathy, pulmonary thromboembolism), renal failure, hemolytic uremic syndrome, and renal thrombotic microangiopathy. Ataxia, encephalopathy, dementia, and psychosis can be reported [76,77,78,79,81]. 

Laboratory findings show homocystinuria, homocysteinemia, methylmalonicacidosis, cystathioninemia with cystathioninuria, and decreased serum methionine. Additionally, ketosis, hyperammonemia, hematuria, proteinuria, megaloblastic anemia, thrombocytopenia, and neutropenia are reported [78,79]. 

Standard long-term treatment includes the administration of L-carnitine, antibiotics to regulate intestinal flora, and vitamin B12 supplementation. Prompt and effective treatment of hyperammonemia is crucial for patient prognosis. A low-protein diet, and supplementation of amino acids, vitamins, and minerals are also important [78].

## 5. Other Diseases Causing Microcephaly and Microcephaly Prevention

### 5.1. Virus Zika Syndrome

Some time ago, the Zika virus was associated with a mild infection, but in recent years it has become one of the most studied viruses in the world [82]. The latest research showed an increase in the number newborns with microcephaly whose mothers were infected with ZIKV during pregnancy [83].

The main symptoms associated with the congenital Zika syndrome are congenital microcephaly, a reduction in cerebral volume, ventriculomegaly, cerebellar hypoplasia, parenchymal or cerebellar calcifications, ocular findings in the posterior and anterior segments, abnormal visual function, and fetal akinesia deformation sequence (i.e., arthrogryposis) [4,83,84]. The clinical course of the acute ZIKV infection in children seems very similar to that in adults, with fever (usually low), rash maculopapular, and pruritus. Neurological complications include Guillain–Barré syndrome and meningoencephalitis [82].

Folic acid supplementation may reduce the risk of the disease (inhibitory effect of FA associated with FRα-AMPK signaling, inhibition of ZIKV replication) [4]. Nowadays there is more detailed knowledge available about the Zika virus, and because of that, pediatricians can diagnose earlier, implement the correct treatment, monitor warnings signs for the most severe forms, and especially establish effective preventive measures [82]. Many variants of ZIKV vaccines have been developed over the past years. These include lice attenuated vaccines, inactivated vaccines, and subunit vaccines [85].

### 5.2. Congenital Rubella Syndrome

Congenital rubella syndrome (CRS) is congenital infections caused by rubella virus. Infection in a woman in the first trimester leads to the development of CRS in 90% of newborns. According to WHO, around 100,000 babies are born with CRS worldwide each year [86].

There is a characteristic triad of symptoms in congenital rubella syndrome, including: cataracts, deafness, and congenital heart disease. This triad is considered to be the major criteria for the diagnosis of Congenital Rubella Syndrome in newborns. There are also minor criteria of CRS, such as: microcephaly, splenomegaly, thrombocytopenia, CRS+ prevention developmental delay, and failure to thrive [87].

The diagnosis of CRS is possible when the newborn has two major components of the triad, or one of the triads and one symptom of minor criteria. The detection of rubella IgM antibodies in laboratory tests, or the persistent level of rubella IgG antibodies in the 6–12 month of an unvaccinated newborn and one with the test, also allows for the diagnosis of CRS [87].

Currently, vaccines based on the live attenuated RA 27/3 shackle are used around the world. This vaccine should be given at 9 or 12–15 months of age in combination with the measles and mumps vaccine. Vaccines are designed to prevent CRS [86,87].

### 5.3. Fetal Alcohol Syndrome (FAS)

Alcohol consumed during pregnancy crosses the placenta and can cause Fetal Alcohol Syndrome (FAS) [88]. FAS is one of the major causes of mental retardation. Studies report incidences is estimated at about 0.2 to 1‰ in the United States of America (USA) and between 1 and 3‰ in France [89]. Alcohol consumption at the beginning of pregnancy leads to the fetal alcohol syndrome, characterized by pre- and post-natal growth restriction, microcephaly, mental retardation, behavioral disorders, impaired coordinative ability, dysmorphic features (narrow palpebral fissure, flat middle face, and hypoplastic philtrum), and cardiac defects [89,90]. “The best prevention strategy to reduce the incidence of FAS is the identification and the consultation of women at high risk, such as women with alcohol abuse or alcohol dependency before their pregnancy and women, who were substantially drinking during earlier pregnancies” [90]. 

### 5.4. Folates—Prevention of Microcephaly

Folic acid deficiency causes malformations of the neural tube in the fetus, which may then result in microcephaly in the newborn. To prevent this, it is recommended to supplement with folic acid 5–6 months before planned pregnancy (it is caused by that supplementation 4 mg folic acid daily, it may take 20 weeks to reach red-blood-cell folate levels between 1050 and 1340 nmol/L, optimal for reduction in the neural tube defect risk), and then consume 400 μg of folic acid daily during pregnancy, this will ensure enough of it needed for organogenesis, and reduce the risk of neural tube defects by 50–70% [91,92].

## 6. Conclusions

Microcephaly is one of the significant clinical manifestations in pediatric neurology, which can be a difficult diagnostic problem due to its different etiology [93]. Microcephaly occurs in various types of metabolic diseases such as inborn glycosylation disorders, mitochondrial diseases, peroxisomal disorders, glucose transporter defects, congenital amino acid metabolism disorders (enzymatic and receptor defects), organic acidosis, or lipid metabolism disorders. Such a wide variety of disorders that lead to the occurrence of microcephaly result in the fact that microcephaly, as a symptom accompanying neurometabolic diseases, is part of a complex clinical picture that requires a complete multidisciplinary approach by neurologists, psychiatrists, cardiologists, orthopedists, or gastroenterologists. Neurometabolic disorders are mostly diagnosed in neonates and infants. Neurological symptoms are very common in this group of diseases. The onset of symptoms of neurometabolic disorders often occurs after initially relatively normal or near-normal growth and development. In addition, affected children may have metabolic crises that have particularly adverse effects on the developing nervous system. During metabolic decompensation, patients with neurometabolic disorders present with severe clinical symptoms, including eating disorders, vomiting, seizures, lethargy, and loss of consciousness. Progression of CNS damage and regression in neurodevelopmental milestones are reported [1]. Therefore, it would be crucial to find a way to effectively restore damaged nerve cells. Medical advances over the past decades have made it possible to diagnose metabolic disorders much earlier than in the past, which contributes to faster treatment. As a result, complications of the disease can be prevented more successfully. The development of molecular medicine and genetics gives hope for a better understanding of the disease mechanism of individual syndromes, which creates a new field for research into new treatment methods. Neurometabolic disorders could be treated at three levels typical of a given disease. First option is enzyme replacement therapy. Second, interventions could be applied at the metabolite level whose aim is to reduce flux through the pathway or to replenish substrates. Third, gene therapy would replace the mutated DNA [94]. The introduction of effective causal treatment into clinical practice would have a key impact on the prognosis of diseases that currently result in a significant percentage of deaths before adolescence, or significantly reduce the quality of life of patients.

## Figures and Tables

**Table 1:** The division of diseases into those with congenital and acquired microcephaly.

Congenital Microcephaly	Acquired Microcephaly
Smith–Lemli–Opitz syndrome	
	Methylmalonic acidemia with homocystinuria
CK syndrome—X-linked syndromic intellectual disability disorder characterized by thin body habitus and cortical malformations	GLUT1 deficiency syndrome 1
Asparagine synthetase deficiency (ASD)	Krabbe disease
Neu–Laxova syndrome—Laxova syndrome type 1 and type 2	Pelizaeus–Merzbacher disease (PMD)
Maternal phenylketonuria	Menkes disease
Microcephaly, Amish type (MCPHA)	Cerebral folate deficiency (CFD)
Methylmalonic acidemia with homocystinuria	Rhizomelic chondrodysplasia punctata type 1 (RCDP1)
Dihydropteridine reductase deficiency (phenylketonuria type 2)	Congenital glycosylation disorder type I
Hyperphenylalaninemia with BH4 deficiency due to GTPCH deficiency	Isolated sulfite oxidase deficiency (ISOD)
Methylenetetrahydrofolate reductase deficiency	Molybdenum cofactor deficiency (MoCoD)
	Methylenetetrahydrofolate reductase deficiency
	Neurodevelopmental disorder with or without hyperkinetic movements and seizures due to NMDA receptor dysfunction- NDHMSA
	Neurodevelopmental disorder with progressive microcephaly, spasticity, and brain imaging abnormalities (NEDMISBA) due to mutations in the MFSD2A gene

## References

[1] Karimzadeh P., Beheshti S., Karimzadeh P., Ave S. (2015). Approach to neurometabolic diseases from a pediatric neurological point of view. Iran J. Child Neurol..

[2] Passemard S., Kaindl A.M., Verloes A. (2013). Microcephaly. Handb. Clin. Neurol..

[3] Harris S.H. (2015). Measuring head circumference: Update on infant microcephaly. Can. Fam. Phys..

[4] Simanjuntak Y., Ko H.Y., Lee Y.L., Yu G.Y., Lin Y.L. (2020). Preventive effects of folic acid on Zika virus-associated poor pregnancy outcomes in immunocompromised mice. PLoS Pathog..

[5] McLarren K.W., Severson T.M., duSouich C., Stockton D.W., Kratz L.E., Cunningham D., Hendson G., Morin R.D., Wu D., Paul J.E. (2010). Hypomorphic temperature-sensitive alleles of NSDHL cause CK syndrome. Am. J. Hum. Genet..

[6] Alfadhel M., Alrifai M.T., Trujillano D., Alshaalan H., Al Othaim A., Al Rasheed S., Assiri H., Alqahtani A.A., Alaamery M., Rolfs A. (2015). Asparagine Synthetase Deficiency: New Inborn Errors of Metabolism. JIMD Rep..

[7] Seidahmed M.Z., Salih M.A., Abdulbasit O.B., Samadi A., Al Hussien K., Miqdad A.M., Biary M.S., Alazami A.M., Alorainy I.A., Kabiraj M.M. (2016). Hyperekplexia, microcephaly and simplified gyral pattern caused by novel ASNS mutations, case report. BMC Neurol..

[8] Wang C., He G., Ge Y., Li R., Li Z., Lin Y. (2020). A novel compound heterozygous missense mutation in ASNS broadens the spectrum of asparagine synthetase deficiency. Mol. Genet. Genom. Med..

[9] Ben-Salem S., Gleeson J.G., Al-Shamsi A.M., Islam B., Hertecant J., Ali B.R., Al-Gazali L. (2015). Asparagine synthetase deficiency detected by whole exome sequencing causes congenital microcephaly, epileptic encephalopathy and psychomotor delay. Metab. Brain Dis..

[10] Acuna-Hidalgo R., Schanze D., Kariminejad A., Nordgren A., Kariminejad M.H., Conner P., Grigelioniene G., Nilsson D., Nordenskjöld M., Wedell A. (2014). Neu-Laxova syndrome is a heterogeneous metabolic disorder caused by defects in enzymes of the L-serine biosynthesis pathway. Am. J. Hum. Genet..

[11] Takeichi T., Okuno Y., Kawamoto A., Inoue T., Nagamoto E., Murase C., Shimizu E., Tanaka K., Kageshita Y., Fukushima S. (2018). Reduction of stratum corneum ceramides in Neu-Laxova syndrome caused by phosphoglycerate dehydrogenase deficiency. J. Lipid Res..

[12] Kaur A., Suranagi V., Patil K., Bannur H. (2018). Neu-Laxova Syndrome: An Unusual Association with Kyphosis. Turk. Patoloji Derg..

[13] Shaheen R., Rahbeeni Z., Alhashem A., Faqeih E., Zhao Q., Xiong Y., Almoisheer A., Al-Qattan S.M., Almadani H.A., Al-Onazi N. (2014). Neu-Laxovasyndrome, an inborn error of serine metabolism, is caused by mutations in PHGDH. Am. J. Hum. Genet..

[14] Barekatain B., Sadeghnia A., Rouhani E., Soofi G.J. (2018). A New Case of Neu-Laxova Syndrome: Infant with Facial Dysmorphism, Arthrogryposis, Ichthyosis, and Microcephalia. Adv. Biomed. Res..

[15] Vockley J., Andersson H.C., Antshel K.M., Braverman N.E., Burton B.K., Frazier D.M., Mitchell J., Smith W.E., Thompson B.H., Berry S.A. (2014). Phenylalanine hydroxylase deficiency: Diagnosis and management guideline. Genet. Med..

[16] Waisbren S., Burton B.K., Feigenbaum A., Konczal L.L., Lilienstein J., McCandless S.E., Rowell R., Sanchez-Valle A., Whitehall K.B., Longo N. (2021). Long-term preservation of intellectual functioning in sapropterin-treated infants and young children with phenylketonuria: A seven-year analysis. Mol. Genet. Metab..

[17] Mitchell J.J., Trakadis Y.J., Scriver C.R. (2011). Phenylalanine hydroxylase deficiency. Genet. Med..

[18] Siu V.M., Ratko S., Prasad A.N., Prasad C., Rupar C.A. (2010). Amish microcephaly: Long-term survival and biochemical characterization. Am. J. Med. Genet..

[19] Kelley R.I., Robinson D., Puffenberger E.G., Strauss K.A., Morton D.H. (2002). Amish lethal microcephaly: A new metabolic disorder with severe congenital microcephaly and 2-ketoglutaric aciduria. Am. J. Med. Genet..

[20] Dayasiri K.C., Suraweera N., Nawarathne D., Senanayake U.E., Dayanath B.K.T.P., Jasinge E., Weerasekara K. (2019). GTP-Cyclohydrolase I deficiency presenting as malignant hyperphenylalaninemia, recurrent hyperthermia and progressive neurological dysfunction in a South Asian child—A case report. BMC Pediatr..

[21] Opladen T., López-Laso E., Cortès-Saladelafont E., Pearson T.S., Sivri H.S., Yildiz Y., Assmann B., Kurian M.A., Leuzzi V., Heales S. (2020). Consensus guideline for the diagnosis and treatment of tetrahydrobiopterin (BH4) deficiencies. Orphanet J. Rare Dis..

[22] Saudubray J.M., Garcia-Cazorla A. (2018). An overview of inborn errors of metabolism affecting the brain: From neurodevelopment to neurodegenerative disorders. Dialogues Clin. Neurosci..

[23] Lilleväli H., Pajusalu S., Wojcik M.H., Goodrich J., Collins R.L., Murumets Ü., Tammur P., Blau N., Lilleväli K., Õunap K. (2020). Genome sequencing identifies a homozygous inversion disrupting QDPR as a cause for dihydropteridine reductase deficiency. Mol. Genet. Genom. Med..

[24] Takahashi Y., Manabe Y., Nakano Y., Yunoki T., Kono S., Narai H., Furujo M., Abe K. (2017). Parkinsonism in Association with Dihydropteridine Reductase Deficiency. Case Rep. Neurol..

[25] Porta F., Ponzone A., Spada M. (2016). Long-term safety and effectiveness of pramipexole in tetrahydrobiopterin deficiency. Eur. J. Paediatr. Neurol..

[26] Temple S.E.L., Sachdev R., Ellaway C. (2020). Familial DHCR7 genotype presenting as a verymild form of Smith-Lemli-Opitz syndrome and lethal holoprosencephaly. JIMD Rep..

[27] Gedam R., Shah I., Ali U., Ohri A. (2012). Smith-Lemli-Opitz-syndrome. Indian J. Hum. Genet..

[28] Park J.E., Lee T., Ha K., Ki C.S. (2021). Carrier frequency and incidence estimation of Smith-Lemli-Opitz syndrome in East Asian populations by Genome Aggregation Database (gnomAD) based analysis. Orphanet J. Rare Dis..

[29] Wassif C.A., Kratz L., Sparks S.E., Wheeler C., Bianconi S., Gropman A., Calis K.A., Kelley R.I., Tierney E., Porter F.D. (2017). A placebo-controlled trial of simvastatin therapy in Smith-Lemli-Opitz syndrome. Genet. Med..

[30] Svoboda M.D., Christie J.M., Eroglu Y., Freeman K.A., Steiner R.D. (2012). Treatment of Smith-Lemli-Opitz syndrome and other sterol disorders. Am. J. Med. Genet. C. Semin. Med. Genet..

[31] Stepanow K.P., Liput M. (2018). Rola kwasu dokozaheksaenowego (DHA) w prawidłowym rozwoju I funkcjonowaniu mózgu oraz siatkówki. Zesz. Nauk. Tow. Dr. UJ Nauk. Ścisłe.

[32] Guemez-Gamboa A., Nguyen L.N., Yang H., Zaki M.S., Kara M., Ben-Omran T., Akizu N., Rosti R.O., Rosti B., Scott E. (2015). Inactivating mutations in MFSD2A, required for omega-3 fatty acid transport in brain, cause a lethal microcephaly syndrome. Nat. Genet..

[33] Harel T., Quek D., Wong B.H., Cazenave-Gassiot A., Wenk M.R., Fan H., Berger I., Shmueli D., Shaag A., Silver D.L. (2018). Homozygous mutation in MFSD2A, encoding a lysolipid transporter for docosahexanoic acid, is associated with microcephaly and hypomyelination. Neurogenetics.

[34] Scala M., Chua G.L., Chin C.F., Alsaif H.S., Borovikov A., Riazuddin S., Riazuddin S., Chiara Manzini M., Severino M., Kuk A. (2020). Biallelic MFSD2A variants associated with congenital microcephaly, developmental delay, and recognizable neuroimaging features. Eur. J. Hum. Genet..

[35] Tang M., Gao G., Rueda C.B., Yu H., Thibodeaux D.N., Awano T., Engelstad K.M., Sanchez-Quintero M.J., Yang H., Li F. (2017). Brain microvasculature defects and Glut1 deficiency syndrome averted by early repletion of the glucose transporter-1 protein. Nat. Commun..

[36] Klepper J., Akman C., Armeno M., Auvin S., Cervenka M., Cross H.J., de Giorgis V., della Marina A., Engelstad K., Heussinger N. (2020). Glut1 Deficiency Syndrome (Glut1DS): State of the art in 2020 and recommendations of the international Glut1DS study group. Epilepsia Open.

[37] Klepper J., Leiendecker B. (2007). GLUT1 deficiency syndrome--2007 update. Dev. Med. Child Neurol..

[38] Ohba C., Shiina M., Tohyama J., Haginoya K., Lerman-Sagie T., Okamoto N., Blumkin L., Lev D., Mukaida S., Nozaki F. (2015). GRIN1 mutations cause encephalopathy with infantile-onset epilepsy, and hyperkinetic and stereotyped movement disorders. Epilepsia.

[39] Lemke J.R., Geider K., Helbig K.L., Heyne H.O., Schütz H., Hentschel J., Courage C., Depienne C., Nava C., Heron D. (2016). Delineating the GRIN1 phenotypic spectrum: A distinctgenetic NMDA receptor encephalopathy. Neurology.

[40] Blakes A., English J., Banka S., Basu H. (2021). A homozygous GRIN1 null variant causes a more severe phenotype of early infantile epileptic encephalopathy. Am. J. Med. Genet. A.

[41] Zehavi Y., Mandel H., Zehavi A., Rashid M.A., Straussberg R., Jabur B., Shaag A., Elpeleg O., Spiegel R. (2017). De novo GRIN1 mutations: An emerging cause of severe early infantile encephalopathy. Eur. J. Med. Genet..

[42] Beltran-Quintero M.L., Bascou N.A., Poe M.D., Wenger D.A., Saavedra-Matiz C.A., Nichols M.J., Escolar M.L. (2019). Early progression of Krabbe disease in patients with symptom onset between 0 and 5 months. Orphanet J. Rare Dis..

[43] Yoon I.C., Bascou N.A., Poe M.D., Szabolcs P., Escolar M.L. (2021). Long-term neurodevelopmental outcomes of hematopoietic stem cell transplantation for late-infantile Krabbe disease. Blood.

[44] Wasserstein M.P., Andriola M., Arnold G., Aron A., Duffner P., Erbe R.W., Escolar M.L., Estrella L., Galvin-Parton P., Iglesias A. (2016). Clinical outcomes of children with abnormal newborn screening results for Krabbe disease in New York State. Genet. Med..

[45] Krieg S.I., Krägeloh-Mann I., Groeschel S., Beck-Wödl S., Husain R.A., Schöls L., Kehrer C. (2020). Natural history of Krabbe disease—A nationwide study in Germany using clinical and MRI data. Orphanet J. Rare Dis..

[46] Tümer Z., Møller L.B. (2010). Menkesdisease. Eur. J. Hum. Genet..

[47] Rangarh P., Kohli N. (2018). Neuroimaging findings in Menkesdisease: A rare neurodegenerative disorder. BMJ Case Rep..

[48] Von der Hagen M., Pivarcsi M., Liebe J., von Bernuth H., Didonato N., Hennermann J.B., Bührer C., Wieczorek D., Kaindl A.M. (2014). Diagnostic approach to microcephaly in childhood: A two-center study and review of the literature. Dev. Med. Child. Neurol..

[49] Kapka-Skrzypczak L., Niedźwiecka J., Diatczyk J., Skrzypczak M., Wojtyła A. (2012). Kwas foliowy- skutki niedoboru i zasadność suplementacji. Med. Og. Nauk. Zdr..

[50] Pope S., Artuch R., Heales S., Rahman S. (2019). Cerebral folate deficiency: Analytical tests and differential diagnosis. J. Inherit. Metab. Dis..

[51] Zhang C., Deng X., Wen Y., He F., Yin F., Peng J. (2020). First case report of cerebral folate deficiency caused by a novel mutation of FOLR1 gene in a Chinese patient. BMC Med. Genet..

[52] Lanska D.J. (2012). Cerebral folate deficiency. MedLinkNeurology.

[53] Frye R.Y., Sequeira J.M., Quadros E.V., James S.J., Rossignol D.A. (2013). Cerebral folate receptor autoantibodies in autism spectrum disorder. Mol. Psychiatry.

[54] Shoffner J., Trommer B., Thurm A., Farmer C., Langley W.A., Soskey L., Rodriguez A.N., d’Souza P., Spence S.J., Hyland K. (2016). CSF concentrations of 5-methyltetrahydrofolate in a cohort of young children with autism. Neurology..

[55] Gonzalez-Ortiz C.L., Jaimes Leguizamon S.B., Contreras-Garcia G.A. (2017). Peroxisomal disorder, rhizomelyc chondrodysplasia punctata type 1: Case report. Rev. Chil. Pediatr..

[56] Baroy T., Koster J., Stromme P., Ebberink M.S., Misceo D., Ferdinandusse S., Holmgren A., Hughes T., Merckoll E., Westvik J. (2015). A novel type of rhizomelic chondrodysplasia punctata, RCDP5, is caused by loss of the PEX5 long isoform. Hum. Mol. Genet..

[57] Bams-Mengerink A.M., Koelman J., Waterham H., Barth P.G., Poll-The B.T. (2013). The neurology of rhizomelic chondrodysplasia punctata. Orphanet J. Rare Dis..

[58] Chang I.J., He M., Lam C.T. (2018). Congenital disorders of glycosylation. Ann. Transl. Med..

[59] Péanne R., de Lonlay P., Foulquier F., Kornak U., Lefeber D.J., Morava E., Pérez B., Seta N., Thiel C., Van Schaftingen E. (2018). Congenital disorders of glycosylation (CDG): Quo vadis?. Eur. J. Med. Genet..

[60] Bogdańska A., Tylki-Szymańska A. (2020). Wrodzone zaburzenia glikozylacji białek—Stale powiększająca się grupa chorób metabolicznych. Postępy Biochem..

[61] Abe Y., Aihara Y., Endo W., Hasegawa H., Ichida K., Uematsu M., Kure S. (2021). The effect of dietary protein restriction in a case of molybdenum cofactor deficiency with MOCS1 mutation. Mol. Genet. Metab. Rep..

[62] Scelsa B., Gasperini S., Righini A., Iascone M., Brazzoduro V.G., Veggiotti P. (2019). Mild phenotype in Molybdenum cofactor deficiency: A new patient and review of the literature. Mol. Genet. Genom. Med..

[63] Reiss J., Hahnewald R. (2011). Molybdenum cofactor deficiency: Mutations in GPHN, MOCS1 and MOCS2. Hum. Mutat..

[64] Gropman A. (2021). Molybdenum cofactor deficiency (MoCD): A Rare Genetic Disorder in Newborns. Neurology Reviews.

[65] Durmaz M.S., Ozbakir B. (2018). Molybdenum cofactor deficiency: Neuroimaging findings. Radiol. Case Rep..

[66] Schwahn B.C., van Spronsen F.J., Belaidi A.A., Bowhay S., Christodoulou J., Derks T.G., Hennermann J.B., Jameson E., Konig K., McGregor T.L. (2015). Efficacy and safety of cyclic pyranopterin monophosphate substitution in severe molybdenum cofactor deficiency type A: A prospective cohort study. Lancet.

[67] Lee H.F., Chi C.S., Tsai C.R., Chen H.C., Lee I.C. (2017). Prenatal Brain disruption in isolated sulfite oxidase deficiency. Orphanet J. Rare Dis..

[68] Claerhout H., Witters P., Regal L., Jansen K., Van Hoestenberghe M.R., Breckpot J., Vermeersch P. (2018). Isolated sulfite oxidase deficiency. J. Inherit. Metab. Dis..

[69] Osório M.J., Goldman S.A. (2018). Neurogenetics of Pelizaeus-Merzbacher disease. Handb. Clin. Neurol..

[70] Najafi K., Kariminejad R., Hosseini K., Moshtagh A., Abbassi G.M., Sadatian N., Bazrgar M., Kariminejad A., Kariminejad M.H. (2017). Familial Case of Pelizaeus-Merzbacher Disorder Detected by Oligoarray Comparative Genomic Hybridization: Genotype-to-Phenotype Diagnosis. Case Rep. Genet..

[71] Baron W., Ozgen H., Klunder B., de Jonge J.C., Nomden A., Plat A., Trifilieff E., de Vries H., Hoekstra D. (2015). The major myelin-resident protein PLP is transported to myelin membranes via a transcytotic mechanism: Involvement of sulfatide. Mol. Cell Biol..

[72] Prukop T., Epplen D.B., Nientiedt T., Wichert S.P., Fledrich R., Stassart R.M., Rossner M.J., Edgar J.M., Werner H.B., Nave K.A. (2014). Progesterone antagonist therapy in a Pelizaeus-Merzbachermouse model. Am. J. Hum. Genet..

[73] Massadeh S., Umair M., Alaamery M., Alfadhel M. (2019). A Novel Homozygous Non-sense Mutation in the Catalytic Domain of MTHFR Causes Severe 5,10-Methylenetetrahydrofolate Reductase Deficiency. Front. Neurol..

[74] Balasubramaniama S., Salomons G.S., Blom H.J. (2013). A case of severe methylenetetrahydrofolate reductase deficiency presenting as neonatal encephalopathy, seizures, microcephaly and central hypoventilation. J. Pediatr. Neurol..

[75] Aljassim N., Alfadhel M., Nashabat M., Eyaid W. (2020). Clinical presentation of seven patients with Methylenetetrahydrofolate reductase deficiency. Mol. Genet. Metab. Rep..

[76] Huemer M., Diodato D., Schwahn B., Schiff M., Bandeira A., Benoist J.F., Burlina A., Cerone R., Couce M.L., Garcia-Cazorla A. (2017). Guidelines for diagnosis and management of the cobalamin-related remethylation disorders cblC, cblD, cblE, cblF, cblG, cblJ and MTHFR deficiency. J. Inherit. Metab. Dis..

[77] Hu S., Mei S., Liu N., Kong X. (2018). Molecular genetic characterization of cblC defects in 126 pedigrees and prenatal genetic diagnosis of pedigrees with combined methylmalonic aciduria and homocystinuria. BMC Med. Genet..

[78] Zhou X., Cui Y., Han J. (2018). Methylmalonic acidemia: Current status and research priorities. Intractable Rare Dis. Res..

[79] Carrillo-Carrasco N., Chandler R.J., Venditti C.P. (2012). Combined methylmalonic acidemia and homocystinuria, cblC type. I. Clinical presentations, diagnosis and management. J. Inherit. Metab. Dis..

[80] Grandone E., Martinelli P., Villani M., Vecchione G., Fischetti L., Leccese A., Santacroce R., Corso G., Margaglione M. (2019). Prospective evaluation of pregnancy outcome in an Italian woman with late-onset combined homocystinuria and methylmalonic aciduria. BMC Pregnancy Childbirth.

[81] Carrillo-Carrasco N., Venditti C.P. (2012). Combined methylmalonic acidemia and homocystinuria, cblC type II. Complications, pathophysiology, and outcomes. J. Inherit. Metab. Dis..

[82] Martins M.M., Medronho R.A., Ledo Alves Da Cunha A.J. (2021). Zika virus in Brazil and worldwide: A narrative review. Paediatr. Int. Child. Health..

[83] Oliveira Melo A.S., Aguiar R.S., Amorim M.M., Arruda M.B., Oliveira Melo F., Ribeiro S.T.C. (2016). Congenital Zika Virus Infection: Beyond Neonatal Microcephaly. JAMA Neurol..

[84] Freitas D.A., Souza-Santos R., Carvalho L.M.A., Barrod W.B., Neves L.M., Brasil P., Wakimoto M.D. (2020). Congenital Zika syndrome: A systematic review. PLoS ONE.

[85] Pattnaik A., Sahoo B.R., Pattnaik A.K. (2020). Current Status of Zika Virus Vaccines: Successes and Challenges. Vaccines.

[86] Kaushik A., Verma S., Kumar P. (2018). Congenital rubella syndrome: A briefre view of public health perspectives. Indian J. Public Health.

[87] Begum N.N.F. (2020). Novel facial characteristics in congenital rubella syndrome: A study of 115 cases in a cardiac hospital of Bangladesh. BMJ Paediatr. Open.

[88] Zuccolo L., DeRoo L.A., Wills A.K., Davey Smith G., Suren P., Roth C., Stoltenberg C., Magnus P. (2016). Pre-conception and prenatal alcohol exposure from mothers and fathers drinking and head circumference: Results from the Norwegian Mother-Child Study (MoBa). Sci. Rep..

[89] Hetea A., Cosconel C., Stanescu A.A.M., Simionescu A.A. (2019). Alcohol and Psychoactive Drugs in Pregnancy. Maedica.

[90] Fröschl B., Brunner-Ziegler S., Wirl C. (2013). Prevention of fetal alcohol syndrome. GMS Health Technol. Assess..

[91] Ferrazzi E., Tiso G., Di Martino D. (2020). Folic acid versus 5- methyl tetrahydrofolate supplementation in pregnancy. Eur. J. Obstet. Gynecol. Reprod. Biol..

[92] van Gool J.D., Hirche H., Lax H., De Schaepdrijver L. (2018). Folic acid and primary prevention of neural tube defects: A review. Reprod. Toxicol..

[93] Szczałuba K., Obersztyn E., Mazurczak T. (2006). Małogłowie jako częsty objaw w praktyce klinicznej—Diagnostyka różnicowa z uwzględnieniem etiopatogenezy. Neurol. Dziec..

[94] Willemsen M.A., Harting I., Wevers R.A. (2016). Neurometabolicdisorders: Five new things. Neurol. Clin. Pract..

